# Enhancing patient-centred chiropractic care in Canada: identifying barriers, enablers, and strategies through a qualitative needs assessment

**DOI:** 10.1186/s12998-024-00560-1

**Published:** 2024-11-28

**Authors:** Daphne To, Danielle Southerst, Melissa Atkinson-Graham, Hainan Yu, Gaelan Connell, Crystal Draper, Carol Cancelliere

**Affiliations:** 1 Canadian Memorial Chiropractic College, Toronto, ON Canada; 2 Institute of Health Policy, Management and Evaluation, University of Toronto, Toronto, ON Canada; 3 Women’s College Hospital Institute for Health System Solutions and Virtual Care, Women’s College Hospital, Toronto, ON Canada; 4 Institute for Disability and Rehabilitation Research and Faculty of Health Sciences, Ontario Tech University, Oshawa, ON L1G 0C5 Canada; 5 Canadian Chiropractic Association, Toronto, ON Canada

**Keywords:** Knowledge translation, Implementation science, Patient-centred care, Patient experience, Behaviour change, Quality improvement, Chiropractic

## Abstract

**Background:**

The Canadian Chiropractic Association (CCA) initiated a quality improvement project to develop *best practices aimed at enhancing the patient experience*.

**Objectives:**

(1) Identify and prioritise the key moments in the new patient experience that could be improved by providing chiropractors with focused support and resources; (2) explore views, barriers, and enablers to implementing these *best practices*; and (3) develop recommendations to facilitate the adoption of these practices.

**Methods:**

We conducted a qualitative needs assessment using a human-centred design approach, focused on understanding the needs and experiences of end-users to create tailored solutions. The Theoretical Domains Framework (TDF) was employed to explore chiropractors’ knowledge use and behaviour change, and TDF domains were mapped to Behaviour Change Techniques (BCTs) to develop targeted strategies for addressing identified barriers and enablers. Thirteen chiropractors from across Canada participated in semi-structured interviews and related activities.

**Results:**

The key moments where participants felt they needed the most support were “treatment”, “report of findings”, “informed consent”, “physical examination”, and “before the appointment”. All participants agreed with the *best practices* seed statements. Key barriers included gaps in knowledge, communication skills, and resource availability, particularly in rural areas. Enablers included collaboration with other health professionals, mentorship, and access to practice tools. Recommendations include enhanced training in communication and treatment planning, increased access to resources in rural areas, and fostering collaborative relationships among health professionals.

**Conclusion:**

Understanding the barriers and enablers to implementing best practices can inform targeted strategies to improve patient-centred care in chiropractic practice across Canada.

**Supplementary Information:**

The online version contains supplementary material available at 10.1186/s12998-024-00560-1.

## Introduction

Patient-centred care is fundamental for improving healthcare quality, as it involves delivering care that is guided by patient values, ensuring that the care meets individual preferences and needs [1]. Current evidence highlights the importance of patient-centred care in enhancing patient experience, clinical outcomes, and satisfaction with healthcare services [2]. The concept of the Quintuple Aim provides a comprehensive framework to optimise health system performance [3]. The five aims for delivering high value care, which should be targets of quality improvement initiatives are: enhancing patient experience, improving population health, reducing costs, improving clinician wellbeing, and advancing health equity [3, 4]. Improving patient experience is critical, as it is associated with better health outcomes, greater adherence to recommended treatments, improved patient safety, and decreased healthcare utilisation, such as reduced hospitalisations and readmissions [5, 6]. It is theorised that quality improvement initiatives are unsustainable if clinician burnout (i.e., poor clinician wellbeing) and health disparities remain high; therefore quality improvement initiatives should be planned and developed with the people who will receive and be most impacted by these initiatives, including both clinicians and patients [3, 4]. Clinician wellbeing has been linked to patient outcomes in various healthcare settings. For instance, burnout has been shown to increase the risk of medical errors and reduce patient satisfaction [7]. Therefore, strategies to promote clinician wellbeing are not just beneficial for healthcare professionals, but are critical to achieving patient-centred care and sustainable quality improvement initiatives.

The use of chiropractic services in Canada has been increasing steadily over the years. A scoping review reported that the 12-month use of chiropractic services in Canada increased from 10 to 11.7% between 1994 and 2012 [8]. Additionally, nation-wide polling data from 2019 indicates that approximately 16% of Canadians had used chiropractic care in the past 12 months [9]. While specific data on the number of new patients seen by chiropractors annually across different provinces or in the average chiropractor’s office are not readily available, the upward trend in usage suggests a growing demand for chiropractic care. This increasing utilisation reflects the important role chiropractic services play in addressing musculoskeletal health issues in Canada. Recognising the importance of patient experience in improving healthcare quality, the Canadian Chiropractic Association (CCA) initiated a quality improvement project aimed at developing *best practices to enhance the patient experience* (Additional File 1) [10]. The seed statements are specifically focused on new patients because the CCA aims to enhance the experience during the first three chiropractic visits. This strategic direction has been informed by nation-wide polling data which indicates that these initial appointments are critical to building a strong foundation for the patient-provider relationship and are pivotal in the successful development of patient trust, loyalty, and retention [9]. The first few visits often shape a patient’s long-term perception of care, influence their likelihood of continuing care or recommending services to others, and impact outcomes [11]. This initiative was led by the Patient Experience Task Force (PETF) and the Patient Experience Review Group (PERG). The Patient Experience Task Force (PETF) was created to design a model patient journey aimed at improving the patient experience in chiropractic care. The PETF’s goals included developing tools and strategies to help Canadian chiropractors understand the drivers of patient trust and engagement. This group consisted of a diverse membership including chiropractors, partners of the chiropractic profession (nationally and provincially), and patient representatives. Members were purposefully selected from across Canada to provide a range of perspectives, including clinical practice, research, and education. The PETF was involved in the development of the best practice statements, also known as seed statements, which were designed to enhance the patient experience during the first three chiropractic visits. To generate these seed statements, the CCA conducted a review of the literature on practices or procedures aimed at improving the patient experience across various health disciplines. The initial draft of the seed statements was created by synthesising key practices identified in the literature. These draft seed statements were then reviewed by the PETF. PETF members rated the “appropriateness” and “importance” of each statement using the criteria defined by the RAND/UCLA Appropriateness Method (RAM) [12]. Statements needed to achieve at least 80% consensus for acceptance. If consensus was not reached, the seed statements were revised and re-rated. This iterative process resulted in the finalisation of 57 seed statements, which provide practice guidance aligned with critical moments in the patient journey, particularly during the initial three visits (Additional File 1) [10]. The Patient Experience Review Group (PERG), composed of chiropractic patients from various regions across Canada, volunteered to participate in evaluating the seed statements. Their role was to rate the seed statements based on their perceived importance to the patient journey, ensuring that the statements aligned with patient expectations and experiences. These seed statements represent a collaborative effort among chiropractors, chiropractic patients, and organisational leaders across Canada to identify actions that can enhance the patient experience in chiropractic care, leading to the cultivation of trust, retention, and loyalty between chiropractors and their patients.

To effectively implement these *best practices*, a comprehensive knowledge translation (KT) plan is essential. The Knowledge-to-Action (KTA) model, a widely used process model in implementation science, guides the translation of research into practice [13, 14]. The KTA model consists of two main phases [14]: knowledge creation and the action cycle. The knowledge creation phase involves synthesising knowledge and producing knowledge tools or products, such as the *best practices* seed statements for patient experience [14]. The action cycle includes activities that facilitate the implementation of this knowledge [14]. An important step in the action cycle is assessing barriers and enablers that affect the implementation of knowledge, practices, or behaviours [14]. Conducting a needs assessment is crucial to systematically identify factors that may impact the uptake of these *best practices* among chiropractors and to develop strategies or interventions that promote their adoption.

The objectives of this study were to:

1. Identify and prioritise the key moments in the new patient experience (i.e., critical moments in the journey of meeting and assessing a new patient) that could be enhanced by providing Canadian chiropractors with focused supports and resources.
2. Explore Canadian chiropractors’ perceived views, barriers, and enablers to implementing the *best practices for the patient experience* seed statements.
3. Develop recommendations for strategies to address the identified barriers and enablers, thereby facilitating the implementation of the *best practices for the patient experience* seed statements by chiropractors in Canada.

## Methods

### Rationale

Quality improvement projects, such as this needs assessment, aim to proactively identify areas for improvement, implement evidence-based interventions, and monitor progress to enhance the quality and outcomes of healthcare delivery [15]. Their iterative nature drives continuous learning, refinement, and optimisation of practices to achieve the best possible results for patients [16].

### Design and approach

Human-centred design (HCD) was a core methodological approach in this study to ensure that the implementation strategies were deeply informed by the needs of the endusers—chiropractors [17–19]. HCD is defined as “a systematic innovation process that prioritizes deep empathy for end-user desires, needs and challenges to fully understand a problem in hopes of developing more comprehensive and effective solutions” [20]. This approach centres users in all stages of research and solution development to ensure that designed outcomes are both relevant and practical for all users. As an innovation approach, this HCD presented agile and empirical methods that facilitated rapid data collection and interpretation designed to inform strategic planning and decision making [21–24]. In this study, HCD was employed through various qualitative research methods, including semi-structured interviews, prioritisation exercises, and feedback gathering. These methods were selected to capture the unique experiences, needs, and challenges of chiropractors in their efforts to implement *best practices*. By integrating HCD with the Theoretical Domains Framework (TDF) [25–27] and Behaviour Change Techniques (BCT), [28–30] the study aimed to develop tailored solutions that would align with chiropractors’ real-world contexts and challenges. We reported this study according to the Consolidated Criteria for Reporting Qualitative Research (COREQ) [31].

### Study team

Our multidisciplinary study team comprised individuals with expertise in clinical epidemiology, health services research, health policy, implementation science, medical anthropology, rehabilitation sciences, chiropractic, and teaching. This diverse background informed our approach to study design, data collection, and analysis. The research team had no prior relationship with any study participants, and we consciously reflected on our biases, values, and experiences throughout the research process [32]. Two members of our research team (CD and CC) were part of the PETF and contributed to the development of the seed statements; however, the PETF and PERG were not involved in the data collection or analysis for this study.

### Theoretical framework

We used methods from implementation science and HCD to explore the unmet needs, barriers, and enablers that shape the implementation of the *best practices* seed statements. Implementation science, defined as the “scientific study of methods to promote systematic uptake of research findings and evidence-based practices into routine practice,” [33] provided a robust framework for assessing these factors. HCD in healthcare innovation focuses on identifying unmet needs and developing solutions tailored to specific contextual and relational dimensions [17–19]. Together, these methods offered a comprehensive approach to assessing the needs of Canadian chiropractors in implementing *best practices*.

We applied the Theoretical Domains Framework (TDF) to explore chiropractors’ perceived barriers and enablers to knowledge use and behaviour change. The TDF is a determinant framework used to identify influences on health professional behaviour change, particularly in the implementation of evidence-based practices [13, 27]. The TDF (version 2) covers 84 theoretical constructs based on psychological theories related to behaviour change and comprises 14 domains reflecting influences on behaviour related to capability, opportunity, and motivation [25, 26].

Finally, we used the Theory and Techniques Tool to map the relevant TDF domains to Behaviour Change Techniques (BCTs) [28, 34, 35]. BCTs are defined as “observable, replicable, and irreducible components of an intervention designed to alter or redirect causal processes that regulate behaviour” [36]. The Theory and Techniques Tool links BCTs with their theoretical mechanisms of action, where the strength of the link was determined by literature synthesis and expert consensus [28, 37].

### Participants

#### Eligibility

All Canadian chiropractors who held an active licence in a province or territory and who were currently involved in the delivery of patient care were eligible for this study.

#### Sampling and recruitment

We used a combination of convenience, purposive, and snowball sampling to identify potential participants [38]. To ensure a broad representation of the Canadian chiropractic profession, we complemented these approaches with maximum variation sampling, capturing a wide range of experiences and perspectives by including chiropractors with varying demographics and practice characteristics [38]. Initial recruitment occurred over a four-week period from September to October 2023.

Convenience and purposive sampling were used to recruit chiropractors who were members of the CCA through postings on the CCA social media page and in the CCA biweekly electronic newsletter, which is sent by email to all CCA members (approximately 8200 members, representing over 80% of licensed chiropractors in Canada) [38]. The recruitment postings for the study informed chiropractors that we were seeking Canadian chiropractors to provide input on initiatives to improve the quality of chiropractic care and enhance patient experiences. For non-CCA members, purposive and snowball sampling were used [38]. Research team members identified non-CCA participants from their professional networks and obtained consent to share their contact information with the research coordinator. The research coordinator then contacted non-CCA members to provide further information about the study. During interviews, non-CCA participants were asked if they could recommend other non-CCA chiropractors, and with consent, the recruitment process was repeated. We acknowledge potential selection bias in our recruitment of non-CCA members, as they were initially identified through our professional networks. To mitigate this, we employed snowball sampling to broaden the participant pool and ensured that non-CCA members were part of the same population as CCA members (i.e., actively practicing chiropractors in Canada).

A recruitment survey was used to collect demographic and practice information before participants were confirmed for interviews (Additional File 2). While this additional step may have impacted willingness to participate, the survey was used to protect participants’ privacy and to ensure that personal information was not shared without explicit consent. The research coordinator (DS) de-identified the collected survey information. We reviewed the de-identified list of survey respondents to select participants based on variation in geographic location, years in practice, age, gender, Indigenous identity, ethnicity, clinical setting, and hours worked per week. The research coordinator contacted selected participants by telephone or email to provide more information about the study and to obtain informed consent. To facilitate snowball sampling, participants were asked to identify additional chiropractors who were not CCA members but might be interested in participating. These chiropractors were contacted using the same strategy outlined above.

#### Description of sample

**Fig. 1:**
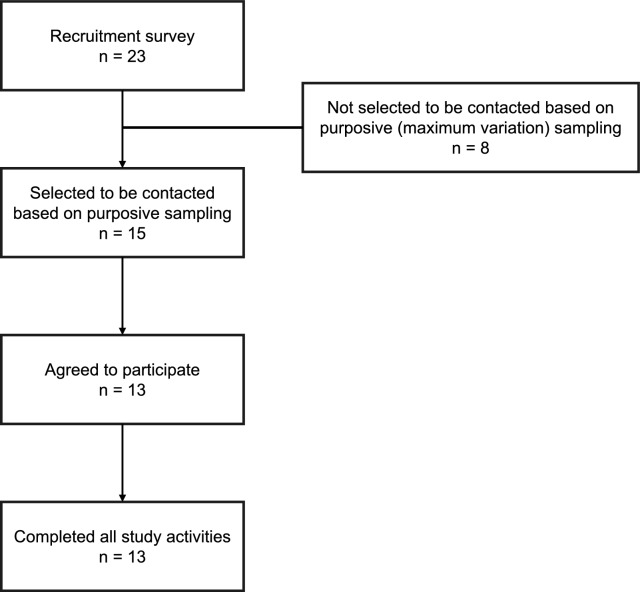
Recruitment and sampling of participants

Twenty-three chiropractors completed the recruitment survey and consented to being contacted about the study. Of these, 15 chiropractors were selected and approached for participation, and 13 consented to participate (Fig. 1). Participants were selected based on their self-reported demographic and practice characteristics from the recruitment survey to ensure a broad representation of the Canadian chiropractic profession. All participants completed the study activities (i.e., prioritisation exercise, interview, and validation/feedback).

Participant demographic and practice characteristics are shown in Table 1. Participants ranged in age from 26 to 67 years (median 37 years), with six identifying as women and ten as White. Two participants identified as Indigenous. Participants practiced in various regions across Canada: four from Ontario, two from Québec, two from Newfoundland and Labrador, and one each from Alberta, British Columbia, Manitoba, Northwest Territories, and Nova Scotia. Eleven of the 13 participants were members of the CCA. Years of practice ranged from 1 to 43 years (median 10 years). Most participants practiced in a major city (6) or a town or smaller regional city (6). Participants commonly practiced in interdisciplinary settings, with eight working alongside complementary and alternative medicine providers and six working alongside other rehabilitation providers. Five participants practiced in more than one setting. Hours worked per week varied, with 10 participants working 21 or more hours per week.

**Table 1:** Participant demographic and practice characteristics (*n* = 13)

	*n*(%)	Median (IQR)
Age (years)		37.0 (30.5; 52.0) Min: 26; Max: 67
*Gender*		
Man	7 (53.8%)	
Woman	6 (46.2%)	
Indigenous identity	2 (15.4%)	
*Ethnicity*		
White	10 (76.9%)	
Other	3 (23.1%)	
*Province of practice*		
Alberta	1 (7.7%)	
British Columbia	1 (7.7%)	
Manitoba	1 (7.7%)	
Newfoundland and Labrador	2 (15.4%)	
Northwest Territories	1 (7.7%)	
Nova Scotia	1 (7.7%)	
Ontario	4 (30.8%)	
Québec	2 (15.4%)	
CCA member	11 (84.6%)	
Years of experience		10.0 (3.5; 25.5) Min: 1.0; Max: 43.0
*Community*		
Rural/remote (population ~ 1000—10,000)	1 (7.7%)	
Town or smaller regional city (population ~ 10,000—100,000)	6 (46.2%)	
Large city (population > 100,000)	6 (46.2%)	
Clinical Setting^a^		
Solo discipline (multiple chiropractors or one chiropractor, including mobile practices)	5 (26.3%)	
Interdisciplinary rehabilitation (chiropractic offered alongside other rehabilitation disciplines such as physiotherapy or occupational therapy)	6 (31.6%)	
Interdisciplinary CAM (chiropractic offered alongside complementary and alternative therapies such as osteopathy, naturopathy, homeopathy and/or massage therapy)	8 (42.1%)	
*Hours worked per week*		
11–20	3 (23.1%)	
21 +	10 (76.9%)	

*CAM* complementary and alternative medicine, *IQR* interquartile range, *Max* maximum value, *Min* minimum value

^a^Five participants reported more than one practice setting

### Data collection and analysis

#### Objective 1:

Identify and prioritise the key moments in the new patient experience

Participants were provided with a copy of the *best practices* seed statements via email, which included a total of 57 seed statements covering seven key moments of *best practices*, with 2–18 seed statements each. Participants first completed a prioritisation exercise, [22] where they identified and ranked the top three key moments in patient care that required the most support to enhance the overall patient experience. They were also asked to rank the seed statements from those they agreed with the most to those they agreed with the least for each of the seven key moments of *best practices*. This prioritisation exercise provided preliminary insight into which *best practices* seed statements resonated most and least with participants. The exercise allowed the research team to pinpoint areas of care that were most critical according to the chiropractors themselves, aligning with HCD’s emphasis on user-driven insights. Participants’ responses to the best practices seed statements were reviewed by the research team before each interview.

#### Objective 2:

Explore Canadian chiropractors’ perceived views, barriers, and enablers to implementing the *best practices* seed statements.

To gain deeper insights into how chiropractors perceived these *best practices* in their practice, we conducted semi-structured, one-on-one interviews lasting approximately 60 minutes with each participant. Each interview was conducted by two members of the research team: one lead interviewer with experience in qualitative research and conducting interviews (DT, MAG, CC), and one note-taker (DS, HY). Interviews were conducted via a videoconferencing platform (Zoom Video Communications, San Jose, California, USA). Given the small sample size, interviews were not recorded to protect participant anonymity. Instead, a second research team member took notes during each interview. The interviewers and note-takers were familiar with the interview questions because they were involved in developing and reviewing the interview guide. After each interview, the lead interviewer and note-taker met to compare notes, summarising key points and reflecting on the content of the interview.

The interviews focused on understanding the personal needs, values, behaviours, practice perspectives, and experiences shaping how participating chiropractors perceived the *best practices* seed statements. Interview questions were informed by the results of the participant’s prioritisation exercise and guided by an interview guide based on the TDF (Additional File 3). This approach allowed for more directed discussions around the ranked *best practices* seed statements, exploring the barriers and enablers associated with their implementation.

A rapid thematic analysis, guided by HCD methods, [22] was conducted to inductively identify key themes and patterns across the results of the prioritisation exercise and interview data. Following each interview, research team dyads tracked the prioritised seed statements, and distilled the barriers and enablers described by the participant into themes. The teams met at the mid-point of the interviews to discuss emerging themes and patterns from the prioritisation exercise results. This approach to theming was informed by the tenets of reflexive thematic analysis [32]. In this approach, researcher subjectivity is not seen as a threat to interpretation, but as a “resource” for thoughtfully interpreting the data [32]. As a team composed of Canadian chiropractors, we brought a distinct, clinically informed empathy to the interpretation of this data, which facilitated a nuanced understanding and description of the unmet needs, barriers, and enablers experienced by participants. The team engaged in reflexive conversations about the data and its interpretation following each interview, in group collaboration sessions, and throughout manuscript development.

The themes and patterns produced through this process reflect the factors that influenced chiropractors’ behaviours related to improving the patient experience and building trust and rapport with patients. The initial analysis was conducted by the lead interviewer (DT, MAG, CC) for each interview, and the relevant and impactful barriers, enablers, and unmet needs were identified. One member of the research team (DT), trained and experienced in using the TDF, coded each of the identified themes into the most relevant domain(s) of the TDF.

#### Objective 3:

Develop recommendations for strategies to address the identified barriers and enablers

The development of targeted intervention strategies was based on a structured, defined process, which involved mapping TDF domains to BCTs using a validated tool [28, 34, 36, 37]. This process was conducted by one member of the research team (DT) and the entire research team reviewed and reached consensus on the list of TDF domains mapped to BCTs. BCTs were selected if there was confirmed or inconclusive evidence supporting the link for each relevant TDF domain identified, based on what was deemed to be feasible and locally relevant. For each BCT, we developed an initial set of potential strategies that could be used by the CCA, drawing on behaviour change literature and participant suggestions. These strategies aimed to target the identified barriers and leverage enablers to improve the implementation of the *best practices*.

To further refine the proposed strategies, participants engaged in a feedback gathering exercise [22]. During this stage, participants were provided with a summary of the barriers, enablers, and potential strategies. They were then asked to provide feedback on the relevance and practicality of these strategies through an open-text survey. This feedback was important in ensuring that the proposed solutions were not only theoretically sound but also actionable and aligned with the chiropractors’ needs. The HCD approach was iterative, involving participants at multiple stages of the study to ensure that their voices were central to the decision-making process. This approach fostered a dynamic, participatory process that enhanced the practicality and relevance of the solutions developed.

#### Knowledge user involvement

The results were presented to a representative from the CCA (CD), who provided feedback on the alignment of the identified strategies with the overall goals and strategic orientation of the CCA. Our developed strategies were then mapped to the CCA’s ongoing and planned initiatives to identify opportunities to address the needs of chiropractors through additional initiatives. This comprehensive approach ensured that the recommendations were practical, feasible, and aligned with the needs of chiropractors, aiming to enhance patient-centred care in chiropractic practice across Canada.

## Results

### Objective 1:

Identify key moments in a patient’s journey where support was needed

**Fig. 2:**
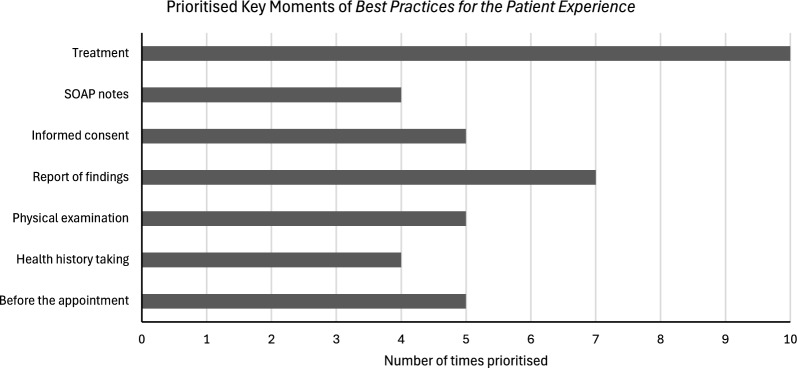
Prioritised key moments of *Best Practices for the Patient Experience.* *One participant prioritised four key moments

Chiropractors in our study most frequently prioritised “treatment” as the critical moment in the patient experience journey, as defined by the CCA *best practices*, that could benefit from targeted support, with 10 participants selecting this key moment (Fig. 2). Other frequently prioritised key moments included “report of findings” (7 participants), “informed consent” (5 participants), “physical examination” (5 participants), and “before the appointment” (5 participants).

### Objective 2:

Explore views, barriers, and enablers to implementing the *best practices* seed statements

All participating chiropractors agreed that the *best practices* seed statements were relevant to chiropractic practice. Many chiropractors expressed difficulty in ranking the *best practices* because they perceived many of the statements as equally important.

Barriers: We identified barriers across eight TDF domains: knowledge; skills; social/professional role and identity; beliefs about capabilities; beliefs about consequences; memory, attention and decision processes; environmental context and resources; and behavioural regulation (Table 2). Early career chiropractors often highlighted gaps in knowledge, particularly in communication and physical examination techniques, as well as challenges in accessing trustworthy information (*TDF domains: knowledge; skills; environmental context and resources*). Some chiropractors expressed a lack of confidence in performing the *best practices* (*TDF domain: beliefs about capabilities*). Environmental barriers such as time constraints, economic pressures, and the availability of resources in rural and remote communities were also noted (*TDF domain: environmental context and resources*).

Additionally, some chiropractors expressed an underutilisation of the biopsychosocial model, noting that it was unclear how to implement this approach or how to adapt it to existing practice models (*TDF domains: knowledge; skills; memory, attention and decision processes*). Those who had already established habits and a style of practice under alternative paradigms faced additional challenges in integrating the biopsychosocial model (*TDF domain: behavioural regulation*). Some chiropractors perceived that the divisions within the chiropractic profession which result in variations in practice philosophies, further complicated the consistent implementation of the *best practices* seed statements (*TDF domain: social/professional role and identity*). Some participants also felt that a lack of any negative consequence to not following the *best practices* seed statements may also be a barrier to its implementation (*TDF domain: beliefs about consequences*).

**Table 2:** Barriers to implementing *best practices for the patient experience* seed statements mapped to the Theoretical Domains Framework

Barrier	Knowledge	Skills	Social/professional role and identity	Beliefs about capabilities	Beliefs about consequences	Memory, attention and decision processes	Environmental context and resources	Behavioural regulation
Uncertainty in where to find trustworthy information for patient care	X						X	
Lack of knowledge and skills on current recommendations for managing non-musculoskeletal or chronic health conditions	X	X						
Lack of training on effective communication skills	X	X						
Lack of training on physical examination skills	X	X						
Lack of culturally relevant resources and training to develop cultural competency	X	X					X	
Lack of understanding on how best to implement the biopsychosocial model	X	X				X		
Lack of formal process to monitor patient trust and experience								X
Having established habits in practice that do not align with patient-centred care								X
Time constraint within clinical encounters							X	
Economic conditions							X	
Geographical location (remote or rural) and the lack of available health services hindering collaboration							X	
Viewing structured education handouts as cookie cutter and not well-received by patients							X	
Perception of confidence and mastery in practice if chiropractors are busy and have financial success				X				
Lack of confidence in being able to perform *best practices* seed statements due to lack of confidence as a chiropractor				X				
Lack of accountability and visibility of negative consequences if not following *best practices* seed statements					X			
Divisions within the chiropractic profession, resulting in variation in practices			X					
Inconsistency across profession in language used by chiropractors			X					

Enablers: We identified enablers corresponding to ten TDF domains: knowledge; skills; social/professional role and identity; beliefs about capabilities; intentions; goals; memory, attention and decision processes; environmental context and resources; social influences; and behavioural regulation (Table 3). Many chiropractors reported that they endeavour to deliver patient-centred care and that the *best practices* seed statements align with this model of care (*TDF domains: beliefs about capabilities; intentions; goals*). More generally, chiropractors shared that they would benefit from resources on culturally safe and appropriate chiropractic care to enhance patient-centricity (*TDF domains: knowledge; skills; environmental context and resources*). Training materials/courses, access to educational resources, and scripts for clear messaging were perceived as helpful for improving communication with patients (*TDF domains: knowledge; skills; environmental context and resources; memory, attention and decision processes*). Practice tools and resources provided by chiropractic associations were noted as enablers for implementing the *best practices* seed statements (*TDF domains: knowledge; skills; environmental context and resources; social/professional role and identity*). Collaboration with other health professionals was identified as an additional source of support (*TDF domain: social influences*). Participants, particularly early career chiropractors, highly valued mentorship from experienced chiropractors (*TDF domains: knowledge; skills; social influences*) and staying updated with best practices through journals and social media (*TDF domains: knowledge; skills; environmental context and resources*). Participants also felt that receiving feedback on their current practices would help enhance their delivery of patient-centred care (*TDF domain: behavioural regulation)*.

**Table 3:** Current and perceived enablers to implementing *best practices for the patient experience* seed statements mapped to the Theoretical Domains Framework

Enabler	Knowledge	Skills	Social/professional role and identity	Beliefs about capabilities	Intentions	Goals	Memory, attention and decision processes	Environmental context and resources	Social influences	Behavioural regulation
*Current enablers*										
Scripts to ensure clear and consistent messaging to patients	X	X					X			
Collaboration with other health professionals to enable appropriate patient referrals									X	
Receiving mentorship from more experienced chiropractors to develop communication and patient management skills	X	X							X	
Staying up to date with best practices/research through journals research reviews	X							X		
Staying up to date by connecting with other clinicians and researchers on social media	X							X		
Use of practice tools and resources provided by chiropractic associations	X	X						X		
Use of electronic health records to improve efficiency and personalise patient interactions								X		
Belief in importance of aligning practice with the latest research	X				X					
Belief in importance of understanding patient preferences		X			X					
Values reflecting on and learning from previous experiences and own clinical practice					X					
Confidence in being able to perform *best practices* seed statements				X						
Strives to create a connection with patients to establish rapport						X				
*Perceived enablers*										
Training and practice on how to communicate effectively with patients (e.g., interviewing)	X	X								
Mental health training for chiropractors	X	X								
Receiving feedback on chiropractors’ current practices to enhance patient-centred care									X	X
Accessible, simple, comprehensive resources for a variety of conditions and clinical tasks (e.g., algorithms, reference book)	X							X		
Practical tools to help chiropractors apply the biopsychosocial model more effectively	X	X						X		
Resources on how to deliver evidence-based, patient-centred care for students and new chiropractors	X	X						X		
Mentorship or courses to help chiropractors learn to grow a patient-centred practice	X	X						X		
Peer support/mentorship									X	
Associations advocating more for chiropractors and the profession			X							

### Objective 3:

Develop recommendations for strategies to address the identified barriers and enablers

We mapped the identified barriers and enablers to the BCTs and developed recommendations to facilitate the implementation of the *best practices* seed statements among chiropractors (Table 4 and Additional File 4). The BCTs most commonly linked were: instruction on how to perform behaviour; demonstration of the behaviour; behavioural practice/rehearsal; prompts and cues; adding objects to the environment; restructuring the physical environment; restructuring the social environment; social support; and goal setting. We propose developing training programs focused on communication skills and the management of diverse patient populations as essential steps. Increasing access to credible information and resources, particularly in rural areas, is crucial to addressing the knowledge gaps identified. Leveraging existing networks and resources will be important for fostering collaborative relationships among health professionals so that they can share best practices. For new graduates and early career chiropractors, we recommend developing mentorship opportunities and networks, allowing them to learn from more experienced peers. Continuous learning and professional development through workshops, seminars, and online courses are recommended to improve the delivery of patient-centred care.

**Table 4:** Potential strategies for implementing *best practices for the patient experience* seed statements informed by the Theoretical Domains Framework mapped to Behaviour Change Techniques

TDF domain	BCT^*^	Potential strategies
Knowledge	4.1 Instruction on how to perform behaviour 6.1 Demonstration of the behaviour	Provide and promote reliable sources for patient care information Provide live training events
Skills	4.1 Instruction on how to perform behaviour 6.1 Demonstration of the behaviour 8.1 Behavioural practice/rehearsal	Provide and promote reliable sources for patient care information Develop training modules and resources focused on patient-centred care Provide live training events with an opportunity to practice skills with peers Offer culturally appropriate toolkits Offer training on culturally safe and appropriate chiropractic care Provide appropriate training in communication Incorporate mental health training into chiropractic education and provide tools to help chiropractors evaluate mental health
Social/professional role and identity	3.1 Social support (unspecified) 6.2 Social comparison 9.1 Credible source	Recognise and reinforce confidence in performing *best practices* seed statements across the profession Foster a unified professional identity that emphasis our common values and prioritises patient-centred care Standardise terminology for communication within the profession Enhance advocacy efforts by professional associations nationally and provincially
Beliefs about capabilities	Goal setting (behaviour) 4.1 Instruction on how to perform behaviour 6.1 Demonstration of the behaviour 15.1 Verbal persuasion about capability 15.3 Focus on past success	Cultivate an understanding that fostering patient experience and trust are important drivers of a successful practice Provide confidence-building programs and mentorship aimed at early career chiropractors Provide mentorship programs and consultations about the unique identity/value of chiropractic
Beliefs about consequences	5.3 Information about social and environmental consequences	Increase awareness of the impact of not adhering to best practices Adopt and endorse the *best practice* seed statements
Intentions	1.1 Goal setting (behaviour)	Reinforce the value of evidence-based practice Encourage patient-centred approaches in all aspects of care Promote a culture of continuous learning and improvement
Goals	Goal setting (behaviour) 1.3 Goal setting (outcome)	Share best practices and success stories to inspire similar approaches
Memory, attention and decision processes	7.1 Prompts/cues 8.4 Habit reversal 11.3 Conserving mental resources	Develop and disseminate scripts for common patient interactions Develop training materials that guide chiropractors in making their own scripts for communicating with patients
Environmental context and resources	3.2 Social support (practical) 7.1 Prompts/cues 12.1 Restructuring the physical environment 12.2 Restructuring the social environment 12.5 Adding objects to the environment	Encourage regular engagement with current research and guidelines Develop low-barrier strategies for interacting with current research and guidelines Develop and distribute clinical resources that are accessible and easy-to-use Develop educational materials and programs for early career chiropractors Promote professional social medial networks for knowledge sharing Increase accessibility and awareness of available resources Develop customised education materials Encourage personalised verbal review of materials in patient handouts Provide training on time management and optimisation of clinic workflows Develop strategies to balance economic pressures with quality patient care Develop referral pathways and networks for chiropractors in regions with limited health services Advocate for and support the adoption of electronic health record
Social influences	3.1 Social support (unspecified) 3.2 Social support (practical)	Establish networks and partnerships for interdisciplinary collaboration Develop mentorship programs for continuous professional development Facilitate peer support networks and mentorship opportunities
Behavioural regulation	2.3 Self-monitoring of behaviour 2.4 Self-monitoring of outcomes of behaviour 8.2 Behaviour substitution 8.3 Habit formation	Develop resources to encourage reflection and self-assessment Establish a system for regular feedback and peer review Develop and implement systems for feedback and monitoring of patient experiences Provide a list of metrics and indicators for monitoring patient satisfaction and experience Monitor patient experience at provincial or national level

*TDF* theoretical domains framework, *BCT* behaviour change technique

^*^BCTs selected if there was confirmed or inconclusive evidence supporting the link for each relevant TDF domain identified and based on what was deemed to be feasible and locally relevant

## Discussion

We conducted a needs assessment with 13 chiropractors across Canada to develop targeted recommendations for implementing the *best practices* seed statements. The prioritisation exercise revealed that “treatment”, “report of findings”, “informed consent”, “physical examination”, and “before the appointment” were the key moments where participants felt they needed the most support. All participating chiropractors agreed that the *best practices* seed statements were relevant and perceived them as critical to enhancing chiropractic practice. The interviews highlighted that chiropractors experienced barriers in their knowledge and skills when trying to implement the *best practices*, highlighting the need for access to credible resources and enhanced training and support to develop or maintain skills in the diagnosis and management of complex musculoskeletal conditions in diverse patient populations. Notable challenges also arose from environmental factors, such as time constraints, economic pressures, and the availability of resources in rural areas. Additionally, the underutilisation of the biopsychosocial model presents a significant barrier, especially for practitioners who have established habits and practices under different paradigms. Mentorship, particularly for early career chiropractors, emerged as a key enabler for implementing best practices. Practice tools and resources from chiropractic associations, along with collaboration with other health professionals and access to culturally appropriate care resources, were also important in patient-centred care.

Conceptual frameworks in the literature have suggested ways to improve patients’ care experiences and enhance clinicians’ ability to deliver patient-centred care [39, 40]. A framework based on the Donabedian model for healthcare improvement proposes that structural domains at the healthcare system and organisational level are foundational components for a patient-centred care model [39]. The framework suggests that organisations play a key role in developing policies, processes, and structures to enable health professionals to deliver patient-centred care [39]. This includes co-designing educational programs, supporting the workforce, and creating structures to measure and monitor patient-centred care [39]. Our current study was aimed at developing strategies to implement the *best practices for the patient experience* seed statements, which were developed by the CCA (i.e., a national organisation). Our findings indicate that chiropractors perceive both healthcare system-level barriers and organisational enablers impacting their ability to implement these seed statements.

Specific to managing people with musculoskeletal pain conditions, a framework designed to enable clinicians to incorporate more patient-centred care principles suggests that the therapeutic relationship is the foundation of care [40]. This framework includes three key principles: a biopsychosocial understanding of the patient’s pain experience, person-focused communication, and supported self-management [40]. Known barriers that health professionals face when trying to build therapeutic alliances and deliver patient-centred care include challenges with applying the biopsychosocial perspective, limited consultation time, lack of knowledge and skills in delivering patient-centred care, and traditional practices and structures [41, 42]. Chiropractors in our study highly valued developing a therapeutic relationship with their patient to improve the patient experience. This is evidenced by their agreement in the relevance of the seed statements to chiropractic practice and their perception that many of the seed statements were equally important.

In line with the findings from broader literature, chiropractors in our study also experienced similar challenges when attempting to deliver patient-centred care. They highlighted the need for strategies that would help them develop the skills to effectively apply the biopsychosocial model, communicate with patients, and provide self-management support to patients. These findings are supported by other studies using the TDF, which has been used to understand health professionals’ behaviour in delivering patient-centred care. TDF-based studies show that clinicians often need tailored interventions to address gaps in knowledge, skills, confidence, and resources in applying evidence-based, patient-centred approaches [43–46]. Similarly, HCD studies emphasise the importance of engaging clinicians in the co-design process to ensure that interventions meet their practical needs [19, 20]. HCD methodologies have been shown to improve the uptake of patient-centred practices by facilitating a deep understanding of user needs and co-developing solutions with health professionals [47].

By integrating insights from both TDF and HCD literature, our study highlights the importance of not only recognising the barriers that chiropractors face in implementing patient-centred care, but also in developing educational tools and interventions that are designed with the users—chiropractors—in mind. Moving forward, a co-designed approach that leverages the experiences of chiropractors and their patients will be crucial in creating strategies that are both effective and practical for the chiropractic profession.

### Strengths

Aligning with the KTA cycle, [14] we conducted a needs assessment to identify factors that impact the uptake of the *best practices* seed statements. A key strength of our approach is the integration of HCD and qualitative research methodologies, which allowed for a thorough exploration of chiropractors’ experiences and perspectives. Other health disciplines have incorporated HCD with implementation science to support the development and tailoring of implementation strategies to meet end-users’ needs, suggesting it is a promising method for improving the implementation of patient-centred care interventions [47].

The TDF guided our semi-structured interviews and analysis, ensuring that our recommendations for improving the implementation of the *best practices* seed statements are theoretically grounded. We followed a systematic approach to mapping the TDF domains to BCTs to develop targeted recommendations, resulting in practical and actionable strategies to address the needs of the participants. The use of theory in intervention design has been shown to increase the likelihood of effectiveness, as the theoretical mechanisms of change can be more systematically evaluated [29]. Furthermore, interventions aimed at improving the management of musculoskeletal conditions, which are underpinned by quality improvement approaches and behaviour change theories such as the TDF, have been shown to result in improvements in various outcomes, including patient experience [48].

Our use of multiple sampling methods enabled the inclusion of a diverse sample of chiropractors from across Canada. In particular, the use of maximum variation sampling ensured a wide range of perspectives were captured, which is important in understanding the diversity of experiences among chiropractors [21].

### Limitations

As with many qualitative studies, our findings are not intended to be generalisable [21]. Our sample may not be representative of all chiropractors in Canada. While we aimed to understand chiropractors’ perceptions of the *best practices* seed statements, the response rate and total number of participants may have limited our ability to capture the full diversity of the chiropractic profession in Canada. The use of a recruitment survey may have potentially limited the number of potential participants that we were able to recruit; however, this decision was made to align with ethical and feasibility considerations. Our study primarily recruited participants from the CCA, which, while reflective of a large portion of Canadian chiropractors (the CCA represents over 80% of licenced chiropractors in Canada), may not fully capture the perspectives of non-members. Our purposive and snowball sampling strategies aimed to capture diverse perspectives by selecting participants based on a combination of demographic and practice characteristics, such as years in practice, type of practice, and patient population, in addition to geographical location. While we did not deliberately underrepresent any jurisdiction, geographic representation was influenced by the willingness of chiropractors in different regions to complete the recruitment survey and consent to being contacted for participation. We acknowledge the underrepresentation of provinces and territories with larger populations (e.g., British Columbia and Alberta) and the absence of participants from certain provinces and territories (e.g., Saskatchewan, New Brunswick, Prince Edward Island, Nunavut, and Yukon).

Additionally, our approach to purposively selecting non-CCA members may have introduced selection bias, [49] as these individuals were recruited through the professional networks of our team. We attempted to mitigate this bias by using snowball sampling to broaden the participant pool and by ensuring that non-CCA members were part of the same practicing population as CCA members (i.e., actively practicing Canadian chiropractors) [49]. Purposive sampling and snowball sampling, while effective for recruiting hard-to-reach populations, have inherent limitations. These methods can result in a lack of representativeness, as participants are often recruited from specific networks, potentially leading to overrepresentation of certain views or experiences. In our study, this may have limited the diversity of perspectives among non-CCA participants. Additionally, snowball sampling relies on participants’ referrals, which can introduce selection bias by recruiting individuals with similar characteristics or experiences to those already in the sample. While we attempted to mitigate these issues by recruiting non-CCA members from diverse practice settings and regions, the potential for bias remains a limitation of the study.

Our design materials and semi-structured interview approach were specifically developed to meet the CCA’s quality improvement needs. Including a larger sample size, particularly with more non-CCA members, and adopting a more exploratory interview approach may have provided additional insights. Additionally, our decision not to record interviews, aimed at protecting participant confidentiality, may have affected the depth and accuracy of the data collected. We mitigated this by conducting debriefing sessions between the interviewer and note-taker immediately after each interview and by having participants review summaries of the results to ensure accuracy.

A limitation of this study is that chiropractic patients were not directly involved in this particular research stage as knowledge users. Although the study’s aim was to understand the perspectives of chiropractors, future research should consider incorporating patients’ views to gain a more comprehensive understanding of how to enhance the patient experience.

### Implications and next steps

Our findings have informed a range of initiatives developed by the CCA and their partners (e.g., Canadian Chiropractic Protective Association) aimed at supporting chiropractors in delivering patient-centred care. These initiatives include programs and resources designed to foster professional development, enhance clinical practices, and improve patient experiences. Examples include a pilot mentorship program to guide early-career chiropractors in achieving their personal and professional goals, as well as workshops focused on key areas such as manual therapy skills, informed consent, communication, and recordkeeping. Continued access to evidence-based resources are also available to enhance chiropractors’ training in various clinical topics. Additionally, resources such as toolkits for creating inclusive intake forms, online learning modules, and validated patient-reported outcome and experience measures are being developed to further support chiropractors in their practice. As part of these efforts, continuing education opportunities (both live and on-demand) are being provided to address diverse topics, including clinical practice management and culturally safe care. The next steps, in line with the KTA cycle, [14] will be to monitor the implementation of these initiatives and evaluate their impact on both patient experience and chiropractors’ perceptions of their ability to deliver patient-centred care.

## Conclusions

Understanding the barriers and enablers to implementing *best practices* in chiropractic care is crucial for developing targeted strategies to enhance patient-centred care. Our study highlights the need for enhanced training in communication and treatment planning, increased access to comprehensive resources, and fostering collaborative relationships among health professionals. By addressing these needs, we can improve the quality of chiropractic care and patient experiences across Canada. Implementing these recommendations may support chiropractors in adopting best practices, ultimately leading to more effective and patient-centred chiropractic care nationwide.

## Supplementary Information

Additional file 1.

Additional file 2.

Additional file 3.

Additional file 4.

## Data Availability

The datasets used and/or analysed during the current study are available from the corresponding author on reasonable request.
