# Supplementary material for: Enhancing patient-centred chiropractic care in Canada: identifying barriers, enablers, and strategies through a qualitative needs assessment
Source: Chiropr Man Therap. 2024 Nov 28;32:37. doi: 10.1186/s12998-024-00560-1 (PMC11605932; doi:10.1186/s12998-024-00560-1)
Supplement: Supplementary file 1 — Additional file 1. [file 12998_2024_560_MOESM1_ESM.pdf]

PATIENT EXPERIENCE TASK FORCE

Consensus Statements on “Best Practices” for the Patient Experience

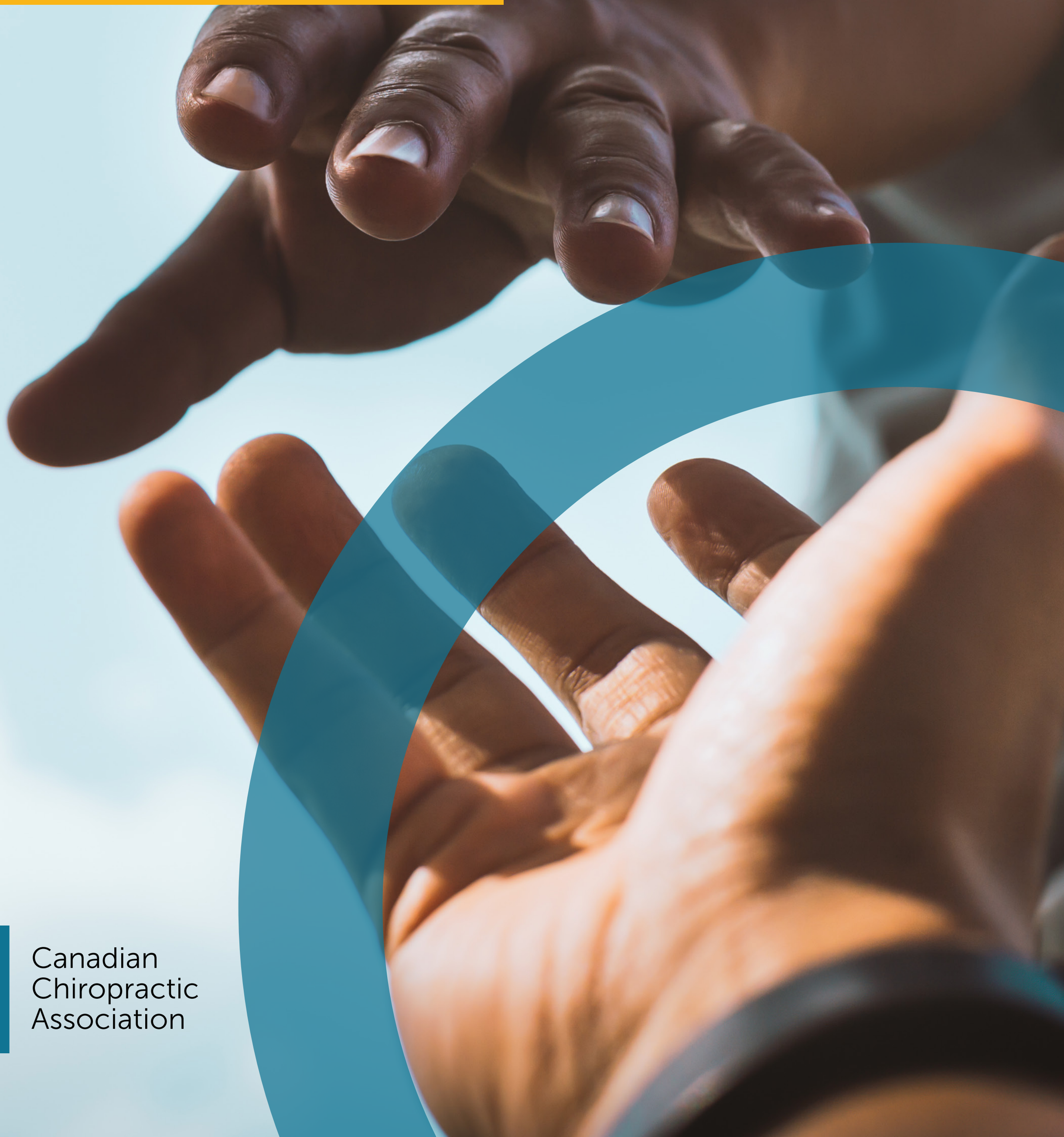

Association chiropratique canadienne

Canadian Chiropractic Association

Introduction

The first three chiropractic appointments have been identified as the deciding factor regarding the successful development of patient trust, loyalty, and retention.

In 2021, the CCA constituted a Patient Experience Task Force (PETF) - a group comprised of engaged chiropractors and chiropractic stakeholders across Canada - to develop a model patient journey.

The following are guiding “seed statements” - a series of best practices opportunities developed to enhance the patient experience. These seed statements were influenced by the Canadian Chiropractic Protective Association’s (CCPA) Roadmap to Care, current evidence, and collective expert opinions about reasonable practices for a positive patient experience during the first three chiropractic visits. They gained overall consensus from the PETF at a level of 80% agreement or higher for appropriateness and importance as well as being uniquely rated by a Patient Experience Review Group (PERG) comprised of chiropractic patients across the country, specifically with respect to importance to the patient journey.

This reference document, along with the other knowledge transfer tools and resources the CCA has developed around patient experience and the model patient journey, aims to support members in their efforts to improve patient engagement and communication while encouraging a more consistent and inclusive initial patient experience. Ultimately, this work directly relates to achieving the CCA’s overall goal of a chiropractor being an integral part of every Canadian’s healthcare team by the end of 2023.

Before the Appointment:

Focusing on the first 3 appointments is crucial in developing patient trust, loyalty and retention. However, the patient experience begins before the first appointment with a chiropractor. The ease of booking an appointment, the clinical environment, and the intake forms and process also contribute to the overall patient journey.

Seed Statement 1:

As a regulated health profession in the healthcare system, chiropractors should support and adhere to all public health policies and initiatives.

Seed Statement 2:

To ensure a positive patient experience while preparing for a chiropractic appointment, a chiropractor should encourage ease of appointment booking(s) for both the initial appointment as well as subsequent appointments (including wait times before appointment, availability of appointment times, and the amount of time it took to arrange an initial appointment).

Seed Statement 3:

To ensure a positive patient experience while preparing for a chiropractic appointment, a chiropractor should encourage a welcoming clinical environment by creating a clean, tidy and professional atmosphere.

Seed Statement 4:

To ensure a positive patient experience while preparing for a chiropractic appointment, a chiropractor should encourage a welcoming clinical environment by ensuring positive interactions with all staff member(s) and practitioner(s).

Seed Statement 5:

To ensure a positive patient experience while preparing for a chiropractic appointment, a chiropractor and/or clinic staff, should communicate with individuals in a way they understand, using clear language, avoiding jargon, and explaining technical terms.

Seed Statement 6:

To ensure a positive patient experience while preparing for, and during an initial chiropractic appointment, a chiropractor should avoid introducing or encouraging a patient into a prolonged treatment term or contract especially before performing a comprehensive assessment (including history and physical examination) and report of findings with the patient.

Health History Taking:

Health history taking is the essential backbone for making an accurate diagnosis and the clinician's first opportunity to get to know their patient and make a positive impression. Thorough history taking allows the clinician to organize the patient's story, filter the relevant information to link to common NMSK disorders by means of clinical reasoning, fully understand the patient's present health status and form a provisional diagnosis.

During a comprehensive health history, questions about the patient's chief complaint, previous health history, family history, red flags and lifestyle factors should be fully explored. Other important components include the following:

Seed Statement 7:

Use of inclusive language in communications, forms and outcome measures (e.g., sex and gender, pronouns including he/him, she/her, they/them, other on intake forms).

Seed Statement 8:

Allowing enough time to listen to the patient carefully and attentively to understand their current status and condition.

Seed Statement 9:

Allowing enough time to obtain an accurate and comprehensive description of symptoms as well as current and past medical history and relevant family history.

Seed Statement 10:

Demonstrating good interpersonal skills with use of appropriate communication methods including verbal and non-verbal cues (i.e., use of open ended questions and questions with options; avoiding leading questions as much as possible; use of open body language; allowing two-way communication and practicing the teach back method[1]).

Seed Statement 11:

Understanding the patient's values and preferences by asking them about their concerns, context, goals and expectations.

Seed Statement 12:

Building a professional relationship with patient by taking time to build a therapeutic relationship early on in the patient journey.

Seed Statement 13:

Building a professional relationship with patient by demonstrating care and concern for patient (treated as an individual person).

Seed Statement 14:

Building a professional relationship by collaborating with the patient to empower them to be a critical and active contributor to their own health.

Seed Statement 15:

Ensure time to document findings accordingly by capturing the full health history and maintaining complete, contemporaneous patient file.

[1] teach back method is a useful way to confirm that the information provided is being understood by getting people to 'teach back' what has been discussed and what instruction has been given.

Physical Examination:

The physical examination can provide a deeper understanding of the patient's health, but also allows the doctor to hone in on the area of chief complaint. The examination should be individualized based on the patient's presenting complaint(s) and the health history you have obtained from them. The history should direct the examination, and the examination should confirm the diagnosis(es) or help exclude relevant differential diagnoses.

Seed Statement 16:

To begin a physical examination, a chiropractor should gain verbal consent to perform the exam and record that it has been received.

Seed Statement 17:

To perform a competent physical examination, a chiropractor should make sure the patient is appropriately dressed for the exam, including gowns and/or shorts as required.

Seed Statement 18:

To facilitate a competent physical examination, a chiropractor should communicate clearly with a patient, using appropriate terminology and signposting.[2]

[2] A signpost is an explicit statement used to inform your patient what you are about to say or do, making it clear to the patient what is going to happen.

Seed Statement 19:

To facilitate a competent physical examination, a chiropractor should allow enough time to perform a thorough physical examination that is personalized and tailored to the patient's chief complaint.

Seed Statement 20:

To facilitate a comprehensive physical examination and to assist in the diagnosis or classification of NMSK conditions, a chiropractor should observe the patient and perform range(s) of motion, neurological screening tests (deep tendon reflexes, motor and sensory testing and/or others, when indicated), orthopedic testing and palpation findings.

Seed Statement 21:

To facilitate a competent physical examination, a chiropractor should ensure the exam is patient-centred by examining with consideration and in response to the individual patient context.

Seed Statement 22:

To facilitate a competent physical examination, a chiropractor should screen patients to identify those with a high likelihood of a serious pathology and/or red flag conditions.

Seed Statement 23:

During the physical examination, a chiropractor should identify the presence of psychosocial factors including yellow flags (i.e., mood/emotions such as depression and anxiety, kinesiophobia/fear-avoidance behaviours and recovery expectations).

Seed Statement 24:

To facilitate a competent physical examination, a chiropractor should discourage radiological and/or diagnostic imaging unless: 1) serious pathology is suspected, 2) there has been unsatisfactory response to appropriate conservative care or unexplained progression of signs and symptoms, or 3) imaging is likely to change management.

Report of Findings:

The report of findings provides an opportunity to discuss the examination findings, diagnosis, prognosis, and the proposed plan of management including the risks and benefits of treatment with the patient.

Seed Statement 25:

To facilitate a thorough report of findings, a chiropractor should provide a patient with a diagnosis with a reasonable, understandable and appropriate explanation using aids, such as models and/or diagrams, the 'teach back' method or the 'chunk and check' method.[3]

Seed Statement 26:

To facilitate a thorough report of findings, a chiropractor should provide a patient with the following: the diagnosis(es); education/information about their condition; management options including reassurance, self-management and the proposed treatment plan (type of treatment, frequency, duration, volume); and the prognosis.

Seed Statement 27:

To facilitate a thorough report of findings, a chiropractor should openly discuss the risks, benefits, and consequences of each treatment option, ensuring the patient understands this includes choosing no treatment or no change to what they are currently doing.

Seed Statement 28:

To facilitate a thorough and accurate report of findings that remains congruent with current best practices, a chiropractor should avoid discussing or describing the patient's condition in fear-based terms that may create, encourage, or justify fear-avoidance behaviour(s) or catastrophizing.

Seed Statement 29:

To facilitate a thorough report of findings, a chiropractor should clearly outline the goals of treatment with specific consideration of the patient's aims, priorities and expectations.

Seed Statement 30:

To facilitate a thorough report of findings, a chiropractor should provide patients with understandable information and guide them toward resources that are accurate, reliable and high quality.

[3] Chunk and check method breaks down information into smaller, more manageable chunks rather than providing it all at once.

Seed Statement 31:

To facilitate a thorough report of findings, a chiropractor should encourage the patient to take an active part in decision making.

Seed Statement 32:

To facilitate a thorough report of findings, a chiropractor should set aside enough time to answer a patient's questions and also ask if they would like a further opportunity to discuss their care.

Informed Consent:

Informed consent is an ongoing process. It is symbolic of the communication between you and the patient about their diagnosis as well as your plan of care and its associated risks.

Seed Statement 33:

To complete a thorough informed consent process, a chiropractor should communicate effectively to confirm patient comprehension (i.e., a patient should understand the recommended care and why they are receiving treatment, the different options and alternatives for care, and the benefits and adverse events of treatment).

Seed Statement 34:

To complete a thorough informed consent process, a chiropractor should determine whether the patient is capable of providing consent (special considerations include the age of patient, separated parents, mental capacity, etc.).

Seed Statement 35:

To complete a thorough informed consent process, a chiropractor should have the patient sign the informed consent form once a diagnosis and plan of management have been communicated and there is confidence that the necessary comprehension and discussion have taken place.

Seed Statement 36:

To complete a thorough informed consent process, a chiropractor should have the patient and doctor sign the informed consent documentation.

Seed Statement 37:

To complete a thorough informed consent process, a chiropractor should practice an ongoing and evolving process and consider updating when appropriate (i.e., treatment plan changes, symptoms and/or diagnosis changes, patient's health status changes or significant changes in the material risks of care, prolonged absence from care, informed consent is stale, and/or if their malpractice protection provider creates a new or updated version of the informed consent form).

SOAP Notes:

SOAP notes should summarize each patient interaction and demonstrate exactly what procedures you have performed and how it helps the patient. These are a crucial part of thorough record keeping practices during a patient's course of care.

Seed Statement 38:

To ensure SOAP notes are thorough and complete, a chiropractor should record entries in a legible, accurate manner with individualized and personal notes capturing the patient's progress and response to care, procedures performed, and advice and recommendations given during each patient encounter.

Seed Statement 39:

To ensure SOAP notes are thorough and complete, a chiropractor should maintain a comprehensive record of care interaction with a patient including the history, physical examination, diagnosis, report of findings, informed consent and treatment notes.

Treatment:

Treatment plans should be a customized, achievable, and measurable care; tailored to the individual patient's needs, values, and preferences.

Seed Statement 40:

To ensure an individualized and comprehensive assessment and treatment plan, a chiropractor should approach patient care using a biopsychosocial model.

Seed Statement 41:

To ensure an individualized and comprehensive treatment plan, a chiropractor should allocate an appropriate amount of time to provide care for the patient.

Seed Statement 42:

To ensure an individualized and comprehensive treatment plan, a chiropractor should practice effective communication and keep patients informed on their condition, treatment plan and recovery expectations.

Seed Statement 43:

To ensure an individualized and comprehensive treatment plan, a chiropractor should practice shared-decision making where treatment plans are agreed upon by both the chiropractor and patient.

Seed Statement 44:

To ensure an individualized and comprehensive treatment plan, a chiropractor should make sure a patient understands the necessity of their participation, and the limitations of your care if they do not do their part.

Seed Statement 45:

To ensure an individualized treatment plan, a chiropractor should listen to and engage patients during treatment.

Seed Statement 46:

To ensure an individualized treatment plan, a chiropractor should address patient questions that arise before, during and after any treatment provided.

Seed Statement 47:

To ensure an individualized treatment plan, a chiropractor should identify and acknowledge patient goals, both short- and long-term.

Seed Statement 48:

To ensure an individualized treatment plan, a chiropractor should proactively monitor and follow-up on how a patient is feeling about their progress, including any dissatisfaction with the care provided.

Seed Statement 49:

To ensure an individualized and comprehensive treatment plan, a chiropractor should use manual therapy as a component of multimodal care in conjunction with other management strategies when appropriate.

Seed Statement 50:

To ensure an individualized and comprehensive treatment plan, a chiropractor should recommend strategies to manage a patient's pain/condition at home, including the prescription of stretches and/or exercises to facilitate active participation and partnership in care.

Seed Statement 51:

To ensure an individualized and comprehensive treatment plan, a chiropractor should advise patients to stay active and gradually increase activity levels, including work involvement when appropriate.

Seed Statement 52:

To ensure an individualized treatment plan, a chiropractor should focus on a patient's return to activity when appropriate.

Seed Statement 53:

To ensure an individualized and comprehensive treatment plan, a chiropractor should perform re-evaluations and/or new clinical assessments at appropriate times during management.

Seed Statement 54:

To ensure an individualized treatment plan, a chiropractor should modify treatment frequency (up to and including discharging a patient) with consideration to when a patient reaches their treatment goals.

Seed Statement 55:

To ensure an individualized treatment plan, a chiropractor should refer a patient appropriately when indicated, including when they present with symptoms that are outside the chiropractic scope of practice.

Seed Statement 56:

To ensure an individualized treatment plan, a chiropractor should refer patients appropriately, including when they fail to improve following a therapeutically relevant trial of care.

Seed Statement 57:

The treatment plan should reflect a chiropractor's competency to remain up to date with current evidence-based practices and clinical guidelines.

Ratings:

Appropriateness
Used to indicate expected health benefit to the patient exceeding the expected negative consequences by a sufficiently wide margin, that it is worth doing, exclusive of cost.

Importance
Used to indicate the value of the procedure or practice to the patient experience.
