# Supplementary material for: Enhancing patient-centred chiropractic care in Canada: identifying barriers, enablers, and strategies through a qualitative needs assessment
Source: Chiropr Man Therap. 2024 Nov 28;32:37. doi: 10.1186/s12998-024-00560-1 (PMC11605932; doi:10.1186/s12998-024-00560-1)
Supplement: Supplementary file 2 — Additional file 2. [file 12998_2024_560_MOESM2_ESM.pdf]

Additional File 2: Recruitment Survey

Survey Instructions: Please complete the following questions. If you do not wish to answer a question, select 'Prefer not to Answer' or indicate 'Prefer not to answer' in the comment box provided. At the end of the survey, you will be asked to provide your email address for the purpose of contacting you to participate in the study. Should you wish to withdraw consent at any time, simply close your browser to discontinue the survey. Only complete surveys will be retained and reviewed.

Part A: Eligibility

1. Are you licensed to practice in Canada?
   - ❑ Yes
   - ❑ No
   - ❑ Prefer not to answer

2. Do you currently provide direct patient care?
   - ❑ Yes
   - ❑ No
   - ❑ Prefer not to answer

Part B. Demographics and Practice Characteristics

1. What is your age in years? (If you prefer not to answer this question, please write 'Prefer not to answer' in the space provided) ____

2. How would you describe your gender identity?
   - ❑ Agender
   - ❑ Gender fluid
   - ❑ Gender queer
   - ❑ Man (includes cis-men, trans-men, and everyone else who identifies as a man)
   - ❑ Non-binary
   - ❑ Woman (includes cis-woman, trans-woman, and everyone else who identifies as a woman)
   - ❑ Other (not listed)
   - ❑ Prefer not to answer

3. Do you identify as an Indigenous person, that is, First Nations (status or non-status), Métis or Inuit or as having Indigenous ancestry?
   - ❑ Yes
   - ❑ No
   - ❑ Do not know
   - ❑ Prefer not to answer

4. Within which ethnic group(s) do you identify? *Select all that apply.*
   - ❑ Arab

- ❑ Black (e.g., African, African-Canadian/American, Afro-Caribbean, Afro-Latinx, etc.)
- ❑ Caribbean (e.g., Antiguan, Bahamian, Barbadian, Bermudan, Carib, Cuban, Dominican, Grenadian, Guadeloupean, Hatian, Jamaican, Kittitian/Nevisian, Martinican, Montserratian, Puerto Rican, St. Lucian, Trinidadian/Tobagonian, Vincentian/Grenadian, West Indian, etc.)
- ❑ Chinese (including Mainland China, Hong Kong, Macau, and Taiwan)
- ❑ Filipino/a/x
- ❑ Indo-Caribbean, Indo-African, Indo-Fijian
- ❑ Japanese
- ❑ Korean
- ❑ Latino/a/x (e.g., Argentinian, Belizean, Bolivian, Brazilian, Chilean, Colombian, Costa Rican, Ecuadorian, Guatemalan, Honduran, Mexican, Nicaraguan, Panamanian, Paraguayan, Peruvian, Salvadorian, Uruguayan, Venezuelan, etc.)
- ❑ North African (e.g., Egyptian, Libyan)
- ❑ Oceania (e.g., Australian, New Zealander, Pacific Islands)
- ❑ South Asian (e.g., Bangladeshi, Pakistani, Indian, Sri Lankan, Punjabi, etc.)
- ❑ Southeast Asian (e.g. Cambodian, Malaysian, Thai, Vietnamese, etc.)
- ❑ West Asian & Middle Eastern (e.g. Afghani, Armenian, Iranian, Iraqi, Israeli, Jordanian, Lebanese, Palestinian, Syrian, Yemeni, etc..)
- ❑ White (e.g., British Isles, French, Western European, Northern European, Eastern European, Southern European, White-Canadian/American/Australian/South African, etc.)
- ❑ Other (not listed)
- ❑ Prefer not to answer

5. How many years of clinical experience do you have? (If you prefer not to answer this question, please write ‘Prefer not to answer’ in the space provided) ____

6. In which province/territory do you primarily practice?
   - ❑ Alberta
   - ❑ British Columbia
   - ❑ Manitoba
   - ❑ New Brunswick
   - ❑ Newfoundland and Labrador
   - ❑ Northwest Territories
   - ❑ Nova Scotia
   - ❑ Nunavut
   - ❑ Ontario
   - ❑ Prince Edward Island
   - ❑ Quebec
   - ❑ Saskatchewan
   - ❑ Yukon
   - ❑ Prefer not to answer

7. Are you a member of the Canadian Chiropractic Association?
   - ❑ Yes
   - ❑ No
   - ❑ Prefer not to answer

8. Which of the following best describes the community where you primarily practice?
   - ❑ Rural/remote region (population: ~1000 to 10,000)
   - ❑ Town or smaller regional city (population: ~10,000 to 100,000)
   - ❑ Major city (urban/metropolitan/suburban) (population: >100,000)
   - ❑ Prefer not to answer

9. What kind of clinical setting do you practice in (check all that apply)?
   - ❑ Solo discipline (multiple chiropractors or one chiropractor, including mobile practices)
   - ❑ Interdisciplinary rehabilitation clinic (i.e. chiropractic offered alongside other rehabilitation disciplines such as physiotherapy or occupational therapy)
   - ❑ Interdisciplinary medical practice (i.e. chiropractic offered alongside physicians or specialists with or without other allied health providers)
   - ❑ Interdisciplinary Complementary and Alternative Medicine clinic (i.e. chiropractic offered alongside complementary and alternative therapies such as osteopathy, naturopathy, homeopathy and/or massage therapy)
   - ❑ Hospital outpatient
   - ❑ Hospital inpatient
   - ❑ Other (not listed)
   - ❑ Prefer not to answer

10. How many hours per week do you spend providing direct patient care?
    - ❑ 0-5 hours
    - ❑ 6-10 hours
    - ❑ 11-20 hours
    - ❑ 21+ hours
    - ❑ Prefer not to answer

Part C. Contact information

1. Would you like the Canadian Chiropractic Guideline Initiative to contact you for the purpose of providing you with additional information about participating in the project titled *Knowledge Translation Plan for Implementing ‘Best Practices for the Patient Experience’ in Chiropractic Practice?* If so, please provide your email address in the question below.
   - ❑ Yes
   - ❑ No

2. If yes, please provide your email address in the space provided:
