# Supplementary material for: Enhancing patient-centred chiropractic care in Canada: identifying barriers, enablers, and strategies through a qualitative needs assessment
Source: Chiropr Man Therap. 2024 Nov 28;32:37. doi: 10.1186/s12998-024-00560-1 (PMC11605932; doi:10.1186/s12998-024-00560-1)
Supplement: Supplementary file 3 — Additional file 3. [file 12998_2024_560_MOESM3_ESM.pdf]

Supplementary File 3: Interview Guide

1. *Introduction*

2. *Participant Background Information*
    - Before we talk about the seed statements, would you tell me a bit more about your practice and your day to day as a chiropractor?
        - Listen for information on years in practice, practice setting, patient population etc.

3. *General Questions*
    - Thinking back to the prioritisation activity:
        - What was your experience going through the exercise?
            - Probing questions: What did you think of the seed statements? What thoughts came to mind about improving the patient experience? Could you share your approach when it came to selecting your top three areas? What about your process when ranking the individual seed statements? What was difficult? Or easy?
            - Probe and or listen to understand if their conception of what chiropractors should be doing to enhance the patient experience is different from seed statements and why

    - As you know, we are interested in learning more about how you go about addressing patient experience and building trust and rapport with your patients. Appreciating that much of this work is done the first 2 to 3 visits, perhaps you could walk us through what those visits look like in your practice and some of the things that you do to build trust with your patients.
        - Probing questions can be used to further explore the top three areas as ranked by the participants
        - Can follow up with talking about a time when things didn't go as expected with respect to patient experience – what did they learn from this experience and how did their practice behaviours change after this

4. *Seed Statement Discussion Questions – Used as probes to drill down into individual practice areas or seed statements that were ranked particularly high or low*

4.1. TDF Knowledge Domain: An awareness of the existence of something (e.g., scientific rationale, procedural knowledge)
- What are your thoughts on the 'best practices for the patient experience' and its components?
    - Probe to understand how the participant approached the prioritization – what informed their selection? What was difficult, or easy?
    - Probe to understand how they related to the statements (e.g. novel, familiar, surprise, agreement, disagreement).
        - Listen for comments regarding absent statements, or what participants expected to find.

- Probe and or listen to understand if their conception of what chiropractors should be doing to enhance the patient experience is different from seed statements and why
- What sources of information do you typically rely on to stay updated with best practices in chiropractic care?
- How confident do you feel in your knowledge of the seed statements you prioritized?

4.2. TDF Skills Domain: An ability or proficiency acquired through practice (e.g., skills development, practice, skill assessment, interpersonal skills)

- What skills or competencies do you think are necessary to implement the 'best practices for the patient experience' effectively?
- What training or education related to the components of the 'best practices for the patient experience' have you received?
    - Probe to understand if there are forms of education, training, or knowledge sharing (e.g. peer support, mentorship) that might be of interest to deliver on patient experience

4.3. TDF Beliefs About Capabilities Domain and Beliefs about Consequences Domain Acceptance of the truth, reality or validity about an ability, talent or facility that a person can put to constructive use (e.g., self-confidence, perceived competence, self-efficacy); Acceptance of the truth, reality, or validity about outcomes of a behaviour in a given situation (e.g., outcome expectancies, anticipated regret)

- If you were asked to implement these practices tomorrow, how would you feel?
    - Probe to understand if participants feel capable/confident/hesitant/unsure if they could implement these practice statements and why. Probe to understand what is shaping the feelings they express.
- What factors might enhance or hinder your confidence in adopting these best practice statements?
- If you were to implement these practices tomorrow, what outcome do you think this would have for your practice?
    - Probe to understand if this is imagined as having positive or negative outcome and why
    - Probe to understand if the participants believes the seed statements will have positive or negative consequences and why

4.4. TDF Environmental Context and Resources Domain: Any circumstance of a person's situation or environment that discourages or encourages the development of skills and abilities, independence, social competence and adaptive behaviour (e.g., material resources, organizational culture/climate, environmental stressors)

- What resources or support do you currently have that would facilitate the implementation of the 'best practices for the patient experience'?
- What environmental or contextual factors may pose challenges to implementing these changes?

4.5. TDF Social Influences Domain: Those interpersonal processes that can cause individuals to change their thoughts, feelings, or behaviours (e.g., social pressure, social norms, group conformity, power, intergroup conflict, social support)

- Are there any individuals or groups who could influence your adoption of the 'best practices for the patient experience'?
    - If relevant, probe to understand why these groups or individuals could influence this behaviour?
        - Probe to understand if related to formal or informal professional networks are they formal or informal groups?
- What formal or informal networks do you rely on for professional guidance or advice?

4.6. TDF Behavioral regulation domain: Anything aimed at managing or changing objectively observed or measured actions (e.g., self-monitoring, breaking habit, action planning)

- Do you monitor the effectiveness of your patient interactions and outcomes? How do you currently monitor or evaluate the effectiveness of your patient interactions and outcomes?
- What strategies do you use to ensure consistency in delivering a positive patient experience?

5. Final Thoughts and Questions

- Is there anything else you would like to share regarding the barriers or facilitators to implementing the 'best practices for the patient experience'?
- What types of resources (including content, format) would you find useful for implementing the best practices?
