# Supplementary material for: Enhancing patient-centred chiropractic care in Canada: identifying barriers, enablers, and strategies through a qualitative needs assessment
Source: Chiropr Man Therap. 2024 Nov 28;32:37. doi: 10.1186/s12998-024-00560-1 (PMC11605932; doi:10.1186/s12998-024-00560-1)
Supplement: Supplementary file 4 — Additional file 4. [file 12998_2024_560_MOESM4_ESM.pdf]

Supplementary File 4: Barriers and enablers to implementing *best practices for the patient experience* seed statements mapped to TDF domains, BCTs, and potential strategies

Barrier	TDF domain	BCT*	Potential strategies
Uncertainty in where to find trustworthy information for patient care	Knowledge	4.1 Instruction on how to perform behaviour	Provide reliable sources for patient care information (e.g., updated resources, algorithms, tools, videos, guidelines).<br><br>Provide 'Welcome Packages' for new CCA members that include a list of reliable sources.<br><br>Engage in strategies to promote these reliable sources (e.g., partner with trusted educators, involve the student chapters of CCA, use of social media).
	Environmental context and resources	7.1 Prompts/cues<br>12.5 Adding objects to the environment	
Lack of knowledge and skills on current recommendations for managing non-MSK or chronic health conditions	Knowledge<br><br>Skills	4.1 Instruction on how to perform behaviour<br>6.1 Demonstration of the behaviour	Provide reliable sources on topics related to signs/symptoms and management of non-MSK and chronic health conditions.<br><br>Provide live training events (in-person, online, or simulation) to provide practical approaches to management.
Lack of training on effective communication skills	Knowledge<br><br>Skills	4.1 Instruction on how to perform behaviour<br>6.1 Demonstration of the behaviour<br>8.1 Behavioural practice/rehearsal	Offer training programs focused on enhancing communication skills in a variety of formats (e.g., workshops, online programs, quick reference guides, videos) with emphasis on various scenarios (e.g., initial visit, subsequent visit).<br><br>Provide live training events (in-person, online, or simulation) to provide chiropractors with the

			opportunity to practice these skills with their peers and experienced clinicians.
Lack of training on physical examination skills	Knowledge<br><br>Skills	4.1 Instruction on how to perform behaviour<br>6.1 Demonstration of the behaviour<br>8.1 Behavioural practice/rehearsal	Offer training programs focused on enhancing physical examination techniques in a variety of formats (e.g., workshops, online programs, quick reference guides, videos).
Lack of culturally relevant resources and training to develop cultural competency	Knowledge<br><br>Skills	4.1 Instruction on how to perform behaviour<br>6.1 Demonstration of the behaviour<br>8.1 Behavioural practice/rehearsal	Offer culturally appropriate toolkits (e.g., self-care handout, patient education video).<br><br>Offer appropriate training on communication and engaging in open and respectful conversations.<br><br>Offer training on culturally safe and appropriate chiropractic care.
	Environmental context and resources	7.1 Prompts/cues<br>12.5 Adding objects to the environment	
Lack of understanding on how best to implement the biopsychosocial model	Knowledge<br><br>Skills	4.1 Instruction on how to perform behaviour<br>6.1 Demonstration of the behaviour<br>8.1 Behavioural practice/rehearsal	Emphasise that patient-centred care and the biopsychosocial model aligns with contemporary healthcare approaches and can significantly enhance patient satisfaction.<br><br>Develop training modules and resources that focus on patient-centred care and the biopsychosocial model and encourage their integration into daily practice.
	Memory, attention and decision processes	7.1 Prompts/cues<br>8.4 Habit reversal	
Lack of formal process to monitor patient trust and experience	Behavioural regulation	2.4 Self-monitoring of outcomes of behaviour	Develop and implement systems for regular feedback and monitoring of patient experiences in individual chiropractic clinics.

			Provide chiropractors with a list of metrics and indicators they can use to monitor patient satisfaction and experience. These metrics may be easier to adopt if they can be incorporated into existing electronical health records or patient management software.<br><br>Monitoring patient experience at the provincial or national level.
Having established habits in practice that do not align with patient-centred care	Behavioural regulation	2.3 Self-monitoring of behaviour<br>8.2 Behaviour substitution<br>8.3 Habit formation	Develop resources to encourage reflection and self-assessment to identify and modify non-patient-centred practices.
Time constraint within clinical encounters	Environmental context and resources	12.1 Restructuring the physical environment<br>12.5 Adding objects to the environment	Provide time management training and training on how to optimise clinic workflows (e.g., provide ideal health history and physical examination forms, provide example timelines for addressing certain visit types).
Economic conditions	Environmental context and resources	12.2 Restructuring the social environment	Develop strategies to balance economic pressures with quality patient care.<br><br>Continue efforts to advocate for the chiropractic profession.
Geographical location (remote or rural) and the lack of available health services hindering collaboration	Environmental context and resources	3.2 Social support<br>12.2 Restructuring the social environment	Develop referral pathways and networks for chiropractors working in regions with limited health services.
Viewing structured education handouts as cookie cutter and not well-received by patients	Environmental context and resources	12.5 Adding objects to the environment	Develop customised education materials to better suit individual patient needs.<br><br>Encourage personalised verbal review of material in handouts and provide training materials on effective educational strategies

			(e.g., chunk and check and teach back methods).
Perception of confidence and mastery in practice if chiropractors are busy and have financial success	Beliefs about capabilities	15.1 Verbal persuasion about capability<br>15.3 Focus on past success	Cultivate an understanding that fostering patient experience and trust are important drivers of a successful practice (e.g., retention, referral).
Lack of confidence in being able to perform *best practices* seed statements due to lack of confidence as a chiropractor	Beliefs about capabilities	1.1 Goal setting (behaviour)<br>4.1 Instruction on how to perform behaviour<br>6.1 Demonstration of the behaviour	Provide confidence-building programs and mentorship aimed at early career chiropractors.<br><br>Consider shifting perspective on what an 'early career' chiropractor is to include a chiropractor in their first 5 years of practice.<br><br>Provide mentorship programs and consultation about the unique identity/value of chiropractic.
Lack of accountability and visibility of negative consequences if not following *best practices* seed statements	Beliefs about consequences	5.3 Information about social and environmental consequences	Increase awareness of the impact of not adhering to best practices.<br><br>Adopt and endorse the *best practice* seed statements (e.g., consider including a mandatory training for membership renewal).
Divisions within the chiropractic profession, resulting in variation in practices	Social/professional role and identity	3.1 Social support<br>6.2 Social comparison<br>9.1 Credible source	Foster a unified professional identity that emphasises our common values and prioritises patient-centred care.
Inconsistency across profession in language used by chiropractors	Social/professional role and identity	9.1 Credible source	Standardise terminology for communication within the profession.

TDF: Theoretical Domains Framework; BCT: Behaviour Change Technique; MSK: Musculoskeletal; CCA: Canadian Chiropractic Association
*BCTs selected if there was confirmed or inconclusive evidence supporting the link for each relevant TDF domain identified and based on what was deemed to be feasible and locally relevant.

Enabler	TDF domain	BCT[*]	Potential strategies
**Current enablers**			
Scripts to ensure clear and consistent messaging to patients	Knowledge<br><br>Skills	4.1 Instruction on how to perform behaviour<br>6.1 Demonstration of the behaviour<br>8.1 Behavioural practice/rehearsal	Develop and disseminate scripts for common patient interactions.<br><br>Develop training materials that guide chiropractors in making their own scripts.
	Memory, attention and decision processes	7.1 Prompts/cues<br>11.3 Conserving mental resources	
Collaboration with other health professionals to enable appropriate patient referrals	Social influences	3.1/3.2 Social support	Establish networks and partnerships for interdisciplinary collaboration.
Receiving mentorship from more experienced chiropractors to develop communication and patient management skills	Knowledge<br><br>Skills	4.1 Instruction on how to perform behaviour<br>6.1 Demonstration of the behaviour	Set up mentorship programs for continuous professional development (e.g., educational outreach visits, academic detailing).
	Social influences	3.1/3.2 Social support	
Staying up to date with best practices/research through journals research reviews	Knowledge	4.1 Instruction on how to perform behaviour	Encourage regular engagement with current research and guidelines.<br><br>Develop low-barrier strategies for interacting with current research and guidelines (e.g., simplified clinical care pathways, 'lunch and learns' or 'coffee chats', brief videos linked through social media).
	Environmental context and resources	3.2 Social support<br>12.5 Adding objects to the environment	
Staying up to date by connecting with other clinicians and researchers on social media	Knowledge	4.1 Instruction on how to perform behaviour	Promote professional social media networks for knowledge sharing for chiropractors across various regions.
	Environmental context and resources	3.2 Social support	
Use of practice tools and resources provided by chiropractic associations	Knowledge<br><br>Skills	4.1 Instruction on how to perform behaviour<br>6.1 Demonstration of the behaviour	Increase accessibility and awareness of available resources sources (e.g., partner with trusted educators, use of social media).

	Environmental context and resources	12.5 Adding objects to the environment	
Use of electronic health records to improve efficiency and personalise patient interactions	Environmental context and resources	12.1 Restructuring the physical environment	Advocate for and support the adoption of electronic health records.<br><br>Develop tools that can integrate into existing electronic health records or patient management software to monitor and enhance patient experience.
Belief in importance of aligning practice with the latest research	Intentions	1.1 Goal setting (behaviour)	Reinforce the value of evidence-based practice.
Belief in importance of understanding patient preferences	Knowledge<br><br>Skills	4.1 Instruction on how to perform behaviour<br>6.1 Demonstration of the behaviour<br>8.1 Behavioural practice/rehearsal	Encourage patient-centred approaches in all aspects of care.<br><br>Provide guidance and training in eliciting patient preferences and incorporating them into clinical decision making
	Intentions	1.1 Goal setting (behaviour)	
Values reflecting on and learning from previous experiences and own clinical practice	Intentions	1.1 Goal setting (behaviour)	Promote a culture of continuous learning and improvement.
Confidence in being able to perform *best practices* seed statements	Beliefs about capabilities	15.1 Verbal persuasion about capability<br>15.3 Focus on past successes	Recognise and reinforce confidence in performing *best practices* seed statements across the profession.
Strives to create a connection with patients to establish rapport	Goals	1.1 Goal setting (behaviour)<br>1.3 Goal setting (outcome)	Share best practices and success stories to inspire similar approaches.
**Perceived enablers**			
Training and practice on how to communicate effectively with patients (e.g., interviewing)	Knowledge<br><br>Skills	4.1 Instruction on how to perform behaviour<br>6.1 Demonstration of the behaviour<br>8.1 Behavioural practice/rehearsal	Provide regular workshops and training sessions (e.g., on motivational interviewing).
Mental health training for chiropractors	Knowledge<br><br>Skills	4.1 Instruction on how to perform behaviour<br>6.1 Demonstration of the behaviour	Incorporate mental health training into chiropractic education and provide tools to help chiropractors evaluate mental health.

		8.1 Behavioural practice/rehearsal	
Receiving feedback on chiropractors' current practices to enhance patient-centred care	Behavioural regulation	2.4 Self-monitoring of outcomes of behaviour	Establish a system for regular feedback and peer review.
	Social influences	3.1/3.2 Social support	
Accessible, simple, comprehensive resources for a variety of conditions and clinical tasks (e.g., algorithms, reference book)	Knowledge	4.1 Instruction on how to perform behaviour	Develop and distribute a wide range of clinical resources that are accessible and easy-to-use.
	Environmental context and resources	12.5 Adding objects to the environment	
Practical tools to help chiropractors apply the biopsychosocial model more effectively	Knowledge<br><br>Skills	4.1 Instruction on how to perform behaviour<br>6.1 Demonstration of the behaviour	Create user-friendly tools and guides for applying the biopsychosocial model.
	Environmental context and resources	12.5 Adding objects to the environment	
Resources on how to deliver evidence-based, patient-centred care for students and new chiropractors	Knowledge<br><br>Skills	4.1 Instruction on how to perform behaviour<br>6.1 Demonstration of the behaviour	Develop educational materials and programs for early career chiropractors.
	Environmental context and resources	12.5 Adding objects to the environment	
Mentorship or courses to help chiropractors learn to grow a patient-centred practice	Knowledge<br><br>Skills	4.1 Instruction on how to perform behaviour<br>6.1 Demonstration of the behaviour<br>8.1 Behavioural practice/rehearsal	Expand mentorship programs and specialised courses (e.g., business training, time management).
	Environmental context and resources	12.5 Adding objects to the environment	
Peer support/mentorship	Social influences	3.1/3.2 Social support	Facilitate peer support networks and mentorship opportunities.
Associations advocating more for chiropractors and the profession	Social/professional role and identity	9.1 Credible source	Enhance advocacy efforts by professional associations nationally and provincially.

TDF: Theoretical Domains Framework; BCT: Behaviour Change Technique; MSK: Musculoskeletal; CCA: Canadian Chiropractic Association
*BCTs selected if there was confirmed or inconclusive evidence supporting the link for each relevant TDF domain identified and based on what was deemed to be feasible and locally relevant.
